# Differences in Nitrogen Metabolism between *Cryptococcus neoformans* and *C. gattii*, the Two Etiologic Agents of Cryptococcosis

**DOI:** 10.1371/journal.pone.0034258

**Published:** 2012-03-27

**Authors:** Popchai Ngamskulrungroj, Yun Chang, Jamin Roh, Kyung J. Kwon-Chung

**Affiliations:** 1 Molecular Microbiology Section, Laboratory of Clinical Infectious Diseases, National Institute of Allergy and Infectious Diseases, National Institutes of Health, Bethesda, Maryland, United States of America; 2 Department of Microbiology, Faculty of Medicine Siriraj Hospital, Mahidol University, Bangkok, Thailand; University of Minnesota, United States of America

## Abstract

Two members of the *Cryptococcus neoformans-gattii* species complex, the etiologic agents of cryptococcosis, can be differentiated by biological, biochemical, serological and molecular typing techniques. Based on their differences in carbon and nitrogen utilization patterns, cost effective and very specific diagnostic tests using D-proline and canvanine-glycine-bromthymol blue (CGB) media have been formulated and are widely used for identification of the two species. However, these methods have yet to be tested for strains with confirmed molecular types to assess the degree of specificity for each molecular type in the two species. We collected global isolates of every major molecular type available and tested their patterns of nitrogen utilization. We confirmed specificity of the CGB test to be 100% regardless of molecular type while the D-proline test yielded 8–38% false negative results in three of the four *C. gattii* molecular types, VGI–VGIII. The utilization pattern of a new set of amino acids: D-alanine, L-tryptophan and L-phenylalanine, showed species specificity comparable to that of D-proline. We discovered that the transcription factor Gat1 (Are1) regulates the utilization of nitrogen differently between *C. neoformans* and *C. gattii* strains. Unlike in *C. neoformans*, expression of the genes encoding glycine decarboxylase complex in *C. gatti* was only partially suppressed by nitrogen catabolite repression in the presence of ammonium. *GAT1* in *C. neoformans* controlled the induction of three of the four genes encoding the glycine decarboxylase complex when glycine was used as the sole nitrogen source while in *C. gattii* its regulation of these genes was less stringent. Moreover, while virulence of *C. neoformans* strains in mice was not affected by Gat1, the transcription factor positively influenced the virulence of *C. gattii* strain.

## Introduction

Two members of *Cryptococcus neoformans-gattii* species complex (*Csc*) are basidiomycetous yeasts that causes cryptococcosis in humans and animals world-wide [1]. Two closely related sister species, *C. neoformans* and *C. gattii* each composed of various subtypes are recognized within the complex [2]. Based on M13 DNA-fingerprinting [3], Amplified Fragment Length Polymorphism (AFLP) [4] and Multilocus Sequence Typing (MLST) [5], [6], [7], *Csc* can be separated into 8 molecular types, VNI-IV and VGI-IV that correlate with four serotypes [8] and 3 varieties [9], [10]. They are, *C. neoformans*: VNI and VNII – serotype A, var. *grubii*; VNIII – serotype AD; VNIV – serotype D, var. *neoformans* and *C. gattii*: VGI-IV – serotype B or C, which was raised to species level in 2002 [11]. In addition, a novel molecular type in *C. neoformans*, VNB, was recently discovered as a unique cryptococcal population in Botswana [12]. Though very closely related, the two species are different in many aspects. *C. neoformans* mainly causes meningoencephalitis in HIV infected patients worldwide [1] except for countries in Far East Asia where cryptococcosis is more common among non-HIV patients [13], [14]. *C. gattii* causes diseases more commonly in non-HIV patients and has gained its importance recently as the causative agent of the cryptococcosis outbreak on Vancouver Island in Canada [15], [16] and the northwest region of USA [17], [18]. The environmental source of *C. neoformans* reported world-wide is usually associated with pigeon guano [10]. *C. gattii*, on the other hand, is known to be associated with a variety of trees, especially Eucalyptus trees [19] and was thought to be restricted to tropical and subtropical areas [20]. Extensive surveys in North and South America, however, revealed that the ecological niche of *C. gattii* has been expanded to areas with temperate climates such as the Pacific Northwest region of USA and Canada [17], [18] or high mountain regions in Colombia [21].

Previous studies have reported that the two species are significantly different in their patterns of nitrogen and carbon utilization. Utilization of creatinine as a nitrogen source was among the first such studies undertaken and found that the strains of serotype A utilize creatinine less efficiently than strains of the other serotypes [22]. An enzymatic assay for creatinine metabolism revealed that creatinine deiminase, which is expressed only in the presence of creatinine, is repressed by ammonia in *C. neoformans* but not in *C. gattii* [23]. Based on these findings, the creatinine bromthymol blue agar was formulated to effectively differentiate the strains of *C. neoformans* from *C. gattii* [23]. D-proline utilization was also found to differ between the two species and D-proline medium was reported to be highly specific for the differentiation of the two species [24], [25]. The enzymatic mechanism of D-proline metabolism, however, has not been studied. *C. gattii* is known to utilize glycine and dicarboxylic acids as carbon sources more effectively than *C. neoformans* [22], [26]. While 88% of *C. gattii* strains utilized glycine, only 20% of *C. neoformans* strains were reported to utilize glycine as a carbon source [26]. A combination of L-canavanine and glycine in the medium allowed 100% differentiation between the two species among the 101 strains of *C. neoformans* and the 70 strains of *C. gattii* tested [27]. A further study from Brazil confirmed the high specificity of the canavanine glycine medium for the differentiation of the two species; only 1 out of 233 strains tested produced an ambiguous result [25].

Utilization of limited nitrogen sources in the host environment is crucial for growth of any pathogenic organism. In order to efficiently regulate the use of nitrogen when abundant, nitrogen catabolite repression (NCR) or nitrogen metabolite repression is induced. NCR ensures the use of preferred nitrogen sources first by suppressing the degradative pathways for secondary nitrogen sources until the readily assimilable nitrogen sources have been exhausted [28], [29]. GATA transcription factors are known to play an important role in regulating the expression of nitrogen-regulated genes [28]. The GATA transcription factor, AreA, Nit2 and Gln3 were among the first and most important transcription factors to be characterized in ascomycetous fungi [28], [29], [30], [31], [32]. It mediates NCR and is required for utilization of secondary nitrogen sources. Apart from its role in nitrogen-regulation, *GAT1* also contributes to the virulence of pathogenic fungi in both humans [32], [33] and plants [34]. Disruption of *GAT1* significantly reduced the virulence of *Candida albicans* [32] and *Aspergillus fumigatus* [33] in a mice model. In *C. neoformans*, the role of *GAT1* was extensively characterized where a relationship was observed between the transcription factor and the genes that are associated with cryptococcal virulence [35], [36]. *GAT1* has been shown to regulate melanin synthesis, capsule production, mating in vitro and virulence in either invertebrate and vertebrate hosts [35]. In spite of the apparent differences between *C. neoformans* and *C. gattii* in the utilization of several nitrogen sources, the role of *GAT1* in regulation of nitrogen assimilation has not been studied.

Although cryptococcosis caused by both species was discovered more than 100 years ago [2], [37], most studies have focused on *C. neformans* and relatively little attention has been paid to *C. gattii*. Comparative biological studies between the two species were mostly carried out prior to the advent of AIDS epidemics [22], [27], [38], [39], [40]. However, after the cryptococcosis outbreak caused by *C. gattii* on Vancouver Island Canada in 1999 [15], *C. gattii* has gained prominence as a primary pathogen. Genetic characterizations of *C. gattii* have been conducted mostly to decipher the virulence traits of the species [41], [42], [43], [44], [45] and not its ability to utilize nitrogen or carbon. Since these biochemical features are crucial for understanding the differences in pathobiology between the two species, we compared the *GAT1* regulation of the amino acid utilization and its effect on virulence.

## Results

### Utilization of nitrogen sources

**Figure 1:**
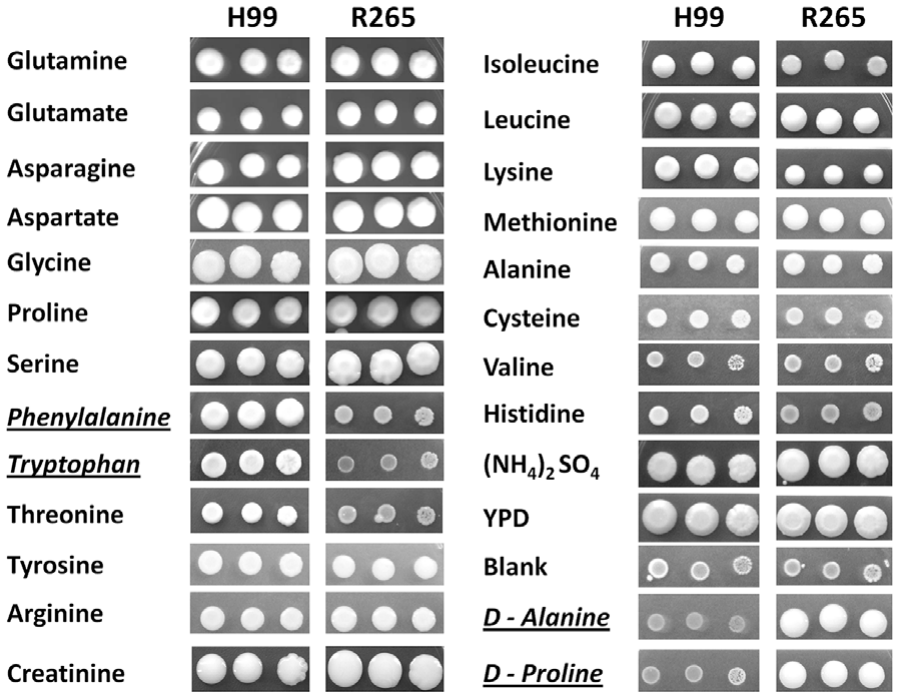
Utilization of nitrogen sources by H99 and R265. 5 µl of cell suspensions at OD_600_nm of 10, 0.1 and 0.001 were spotted on 2% glucose YNB agar with 10 mM of the indicated nitrogen source and incubated for 5–7 days at 30°C. Amino acids differentially utilized by the two species are italicized and underlined.

Initial screening of nitrogen utilization was performed with H99 [46] and R265 [15] as the reference strains of *C. neoformans* and *C. gattii*, respectively, since these two genome sequenced strains are typically used for molecular genetics and pathogenesis studies [1]. Nitrogen sources used in the study included 23 amino acids (see Fig. 1) and ammonium sulfate. Yeast peptone glucose (YPD) agar was used as a positive control for growth and yeast nitrogen base (YNB) with 2% glucose but without amino acids and ammonium sulfate (Fig. 1, Blank) was used as a negative control. As shown in Figure 1, utilization of the nitrogen sources was comparable between the two species except for L-phenylalanine, L-tryptophan, D-proline and D-alanine. Although threonine utilization was also different between the two strains, the difference was not as robust as with the aforementioned four amino acids (Fig. 1). While D-proline and D-alanine were utilized exclusively by R265, L-phenylalanine and L-tryptophan were utilized by H99 significantly better than R265. We randomly selected 67 strains of all the known major molecular types (8 VNI, 3 VNII, 3 VNB, 6 VNIII, 11 VNIV, 8 VGI, 12 VGII, 11 VGIII and 5 VGIV). The molecular types of the strains were confirmed by *URA5*-RFLP and then the strains were tested for utilization of the four amino acids as the source of nitrogen (Table 1). The results were compared with the standard CGB test (Table 1) and the species specificity of the amino acid utilization was summarized (Table 2). The CGB test was species specific, regardless of molecular type. Interestingly, utilization of D-alanine was found to be more species specific than D-proline which has been the second most widely used reagent to differentiate between the two species. While the test involving utilization of D-proline did not yield any false positive results for the *C. neoformans* strains, six of the 36 (17%) *C. gattii* strains were false negative. False negatives were obtained for three of the four *C. gattii* molecular types (VGI–VGIII) with the highest frequency observed among strains of molecular type VGI (38%) followed by VGIII (18%) and VGII (8%). No false negative results were obtained with VGIV strains. As is the case with D-proline, no *C. neoformans* strains utilized D-alanine (Table 2) while 94% (34/36) of the *C. gattii* strains were able to utilize D-alanine as the sole source of nitrogen. Interestingly, false negatives for D-alanine were only obtained for strains of the VGI molecular type (25%). Among the *C. gatti* strains tested, only 6% could utilize L-Phenylalanine while none could utilize L-Tryptophan as a nitrogen source (Tables 1 and 2). Interestingly, the ability to utilize L-Phenylalanine or L-Tryptophan as a nitrogen source was specific to only the VNI, VNII, VNB (serotype A/var. *grubii*; 93% for L-Phenylalanine and 79% for L-tryptophan) and VNIII, the serotype A/D hybrid (100% for either L-Phenylalanine or L-Tryptophan) molecular type strains. Most VNIV strains (82%) poorly utilized L-Phenylalanine or L-Tryptophan as the sole nitrogen source (Tables 1 and 2).

**Table 1:** List of strains and their ability to utilize different nitrogen sources.

Name	Country	Species	Moleculartype	D-Proline	D-Alanine	L-Phenylalanine	L-Tryptophan	CGB
B5763	unknown	*C. gattii*	VGI	−	−	−	−	+
B5765	unknown	*C. gattii*	VGI	+	+	−	−	+
B5788	unknown	*C. gattii*	VGI	+	+	−	−	+
NIH254	unknown	*C. gattii*	VGI	−	−	−	−	+
WM179	Australia	*C. gattii*	VGI	−	+	−	−	+
WM276	Australia	*C. gattii*	VGI	+	+	+	−	+
B4506	Australia	*C. gattii*	VGI	+	+	−	−	+
NIH76	unknown	*C. gattii*	VGI	+	+	−	−	+
475061	Thailand	*C. gattii*	VGII	+	+	−	−	+
B4534	unknown	*C. gattii*	VGII	+	+	−	−	+
H001875	Colombia	*C. gattii*	VGII	+	+	−	−	+
H001941	Colombia	*C. gattii*	VGII	−	+	−	−	+
McBride	Australia	*C. gattii*	VGII	+	+	−	−	+
NIH444	USA	*C. gattii*	VGII	+	+	−	−	+
R265	Canada	*C. gattii*	VGII	+	+	−	−	+
R272	Canada	*C. gattii*	VGII	+	+	−	−	+
Ram002	Australia	*C. gattii*	VGII	+	+	−	−	+
RB1	Canada	*C. gattii*	VGII	+	+	−	−	+
VPB058	Australia	*C. gattii*	VGII	+	+	−	−	+
WM178	Australia	*C. gattii*	VGII	+	+	−	−	+
NIH18	unknown	*C. gattii*	VGIII	−	+	−	−	+
NIH113	unknown	*C. gattii*	VGIII	+	+	−	−	+
NIH409	unknown	*C. gattii*	VGIII	+	+	−	−	+
CN043S	New Zealand	*C. gattii*	VGIII	+	+	−	−	+
CN043W	New Zealand	*C. gattii*	VGIII	+	+	−	−	+
H0078	Colombia	*C. gattii*	VGIII	+	+	−	−	+
H00818	Colombia	*C. gattii*	VGIII	+	+	−	−	+
NIH191	USA	*C. gattii*	VGIII	+	+	−	−	+
NIH312	unknown	*C. gattii*	VGIII	+	+	−	−	+
NIH744	unknown	*C. gattii*	VGIII	−	+	−	−	+
WM175	USA	*C. gattii*	VGIII	+	+	−	−	+
105	unknown	*C. gattii*	VGIV	+	+	−	−	+
LA390	Mexico	*C. gattii*	VGIV	+	+	−	−	+
H001686	Colombia	*C. gattii*	VGIV	+	+	−	−	+
NIH535	unknown	*C. gattii*	VGIV	+	+	−	−	+
WM779	South Africa	*C. gattii*	VGIV	+	+	−	−	+
Bt85	Botswana	*C. neoformans*	VNB	−	−	+	+	−
Bt88	Botswana	*C. neoformans*	VNB	−	−	+	+	−
Bt131	Botswana	*C. neoformans*	VNB	−	−	+	+	−
NIH289	unknown	*C. neoformans*	VNI	−	−	+	+	−
C48	USA	*C. neoformans*	VNI	−	−	+	+	−
H99	USA	*C. neoformans*	VNI	−	−	+	+	−
It743	Italy	*C. neoformans*	VNI	−	−	+	+	−
JP1088	Japan	*C. neoformans*	VNI	−	−	+	+	−
Mal212	Malawi	*C. neoformans*	VNI	−	−	+	+	−
WM148	Australia	*C. neoformans*	VNI	−	−	+	+	−
NIH394	unknown	*C. neoformans*	VNI	−	−	−	−	−
NIH281	unknown	*C. neoformans*	VNII	−	−	+	−	−
NIH286	unknown	*C. neoformans*	VNII	−	−	+	+	−
WM626	Australia	*C. neoformans*	VNII	−	−	+	−	−
CBS132	Japan	*C. neoformans*	VNIII	−	−	+	+	−
KW5a	Kuwait	*C. neoformans*	VNIII	−	−	+	+	−
NIH304	unknown	*C. neoformans*	VNIII	−	−	+	+	−
RKIM364	Germany	*C. neoformans*	VNIII	−	−	+	+	−
SpaE13	Spain	*C. neoformans*	VNIII	−	−	+	+	−
TBS54	India	*C. neoformans*	VNIII	−	−	+	+	−
NIH487	unknown	*C. neoformans*	VNIV	−	−	−	−	−
NIH310	unknown	*C. neoformans*	VNIV	−	−	+	+	−
NIH531	unknown	*C. neoformans*	VNIV	−	−	+	+	−
B3501	USA	*C. neoformans*	VNIV	−	−	−	−	−
B3502	USA	*C. neoformans*	VNIV	−	−	−	−	−
JEC20	USA	*C. neoformans*	VNIV	−	−	−	−	−
JEC21	USA	*C. neoformans*	VNIV	−	−	−	−	−
NIH12	unknown	*C. neoformans*	VNIV	−	−	−	−	−
NIH430	unknown	*C. neoformans*	VNIV	−	−	−	−	−
NIH433	unknown	*C. neoformans*	VNIV	−	−	−	−	−
WM629	Australia	*C. neoformans*	VNIV	−	−	−	−	−

**Table 2:** Utilization of different nitrogen sources according to molecular type.

	Species	Molecular type										
		*C.n.*	*C.g.*	VNI	VNB	VNII	VNIII	VNIV	VGI	VGII	VGIII	VGIV
D-proline	negative	***31***	6	***8***	***3***	***3***	***6***	***11***	3	1	2	0
	positive	***0***	30	***0***	***0***	***0***	***0***	***0***	5	11	9	5
D-alanine	negative	***31***	2	***8***	***3***	***3***	***6***	***11***	2	0	0	0
	positive	***0***	34	***0***	***0***	***0***	***0***	***0***	6	12	11	5
L-Phenylalanine	negative	10	35	1	0	0	0	9	7	12	11	5
	positive	21	1	7	3	3	6	2	1	0	0	0
L-Tryptophan	negative	12	***36***	1	0	2	0	9	***8***	***12***	***11***	***5***
	positive	19	***0***	7	3	1	6	2	***0***	***0***	***0***	***0***
CGB	negative	***31***	0	***8***	***3***	***3***	***6***	***11***	***0***	***0***	***0***	***0***
	positive	***0***	36	***0***	***0***	***0***	***0***	***0***	***8***	***12***	***11***	***5***

*C. n.* = *Cryptococcus neoformans* and *C.g.* = *Cryptococcus gattii*. Bold italic are test giving 100% correlation with either species.

### Regulation of amino acid utilization by *GAT1* is different between the two species

**Figure 2:**
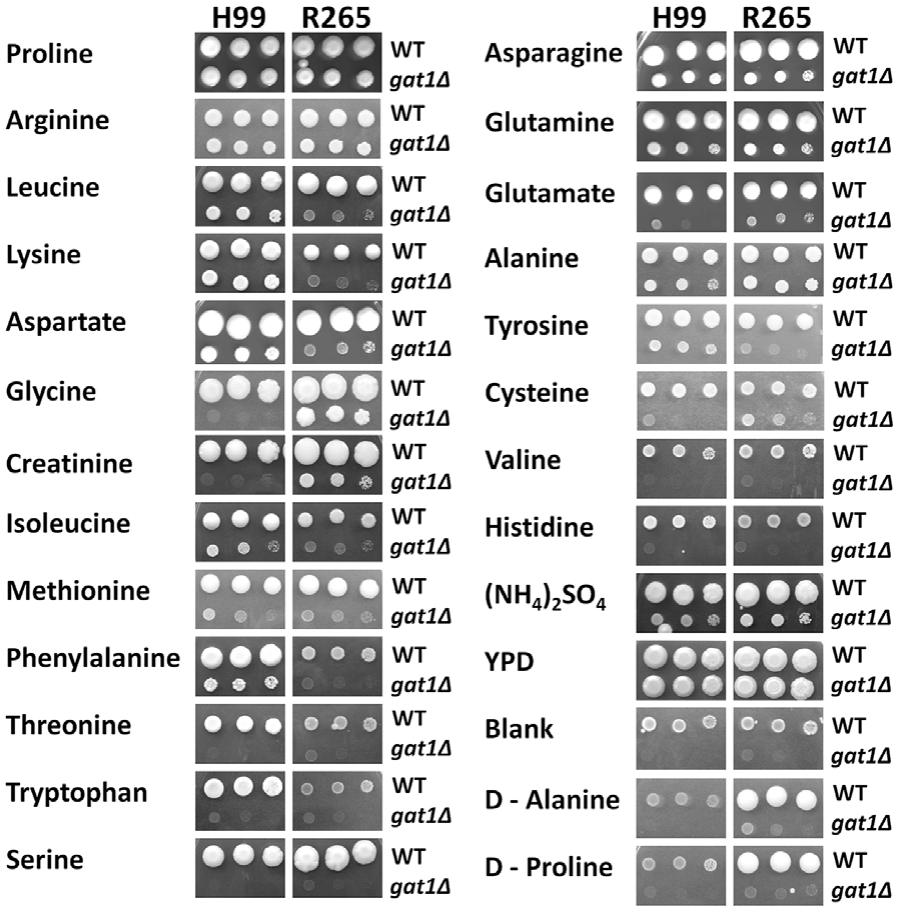
Disruption of *GAT1* reduced ability to utilize all nitrogen sources. Wild type and *gat1Δ* strains were grown for 5–7 days at 30°C on 2% glucose YNB with 10 mM of different nitrogen sources. 5 µl cell suspensions at OD600nm of 10, 0.1 and 0.001 were spotted on the media. YPD and Blank (no nitrogen source) served as positive and negative control respectively.

Previous study on the differences in creatinine metabolism between *C. neoformans* and *C. gattii* [47] suggested that the divergence in nitrogen utilization between the two species could be due to their differences in NCR (Nitrogen Catabolite Repression). Creatinine deiminase, a key enzyme for creatinine metabolism, was found to be repressed in *C. neoformans* but not in *C. gattii* by accumulation of ammonium in the growth medium [47]. Importance of the NCR mechanism in nitrogen utilization has been emphasized by recent studies in *C. neoformans* [35], [36]. *GAT1*, a GATA transcription factor, is required for utilization of all of the tested nitrogen sources via the NCR mechanism [35]. Thus, we identified and disrupted the *GAT1* gene in both species and compared the control of nitrogen utilization by *GAT1*. The *GAT1* gene in R265 was identified as CNBG_0368 based on its sequence similarity with the H99 *GAT1* (CNAG_00193) [36]. As shown in Figure 2, the *gat1Δ* strains of H99 and R265, as representatives of the two species, differed significantly in their ability to utilize various nitrogen sources. Growth of R265*gat1Δ and* H99*gat1Δ* strains was markedly reduced in all nitrogen sources tested except for proline and arginine where only a slight reduction was observed for both species. Unlike *C. neoformans*, only a slight reduction of alanine utilization was observed in *C. gattii*. Growth of R265*gat1Δ* on L-leucine, L-lysine, and L-aspartate was significantly reduced compared to H99*gat1Δ*. On the other hand, *GAT1* disruption caused only slight reduction in the growth of R265 on glycine and creatinine while the *GAT1* disruption in H99 totally abolished its ability to utilize the same nitrogen source (Fig. 2). Complementation of *GAT1* restored the wild type level of ability to utilize different amino acids as the sole nitrogen source (Fig S1).

### The expression levels of genes encoding glycine cleavage enzymes are controlled by nitrogen catabolite repression less stringently in *C. gattii* than in *C. neoformans*

**Figure 3:**
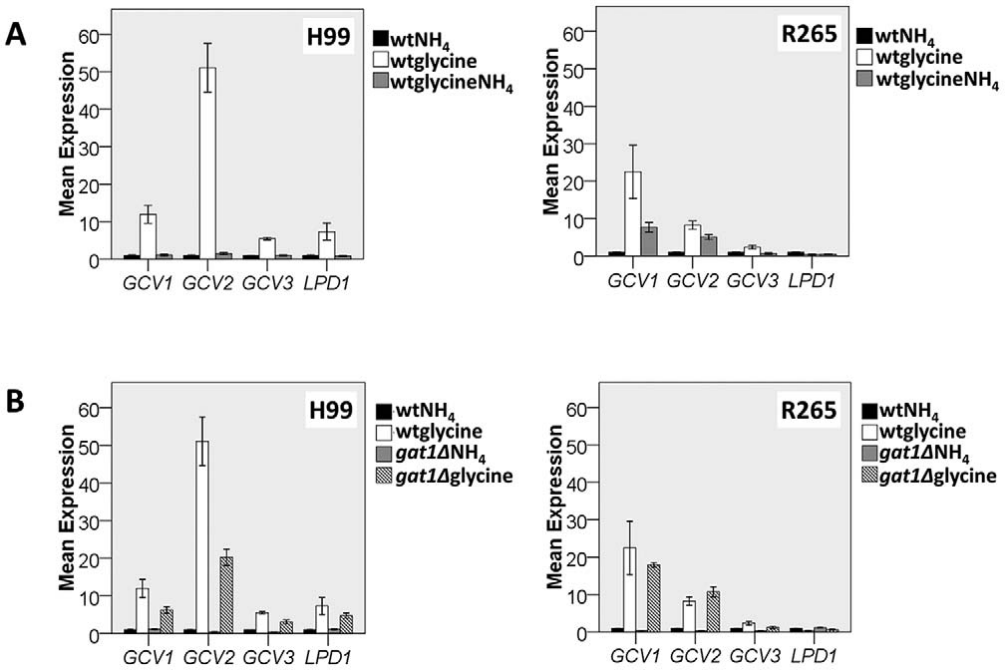
Comparison of expression levels of the genes encoding glycine cleavage enzymes in H99 and R265. RNA levels of genes encoded enzymes in the glycine cleavage decarboxylase complex were determined in cells growing in ammonium sulfate (NH_4_), glycine or glycine plus ammonium sulfate (glycineNH4) as the sole nitrogen source. Expression is presented in folds of wild type levels growing in ammonium sulfate. A) Expression levels of the four genes in the wild type strains. B) Effect of *GAT1* deletion on the expression of glycine decarboxylase complex genes. The experiment was carried out in triplicates. Bars = standard error, wt = wild type.

Since utilization of glycine as both the carbon and the nitrogen source has been used to differentiate between the two species [27], we compared the regulation of glycine utilization between H99 and R265 as representatives of the two species. In *Saccharomyces cerevisiae*, glycine is utilized as a nitrogen source via cleavage by the glycine decarboxylase complex which is controlled by NCR [48]. *C. neoformans* and *C. gattii* both contain the *S. cerevisiae* orthologs encoding 4 subunits of the glycine decarboxylase complex: the T-protein (encoded by *GCV1* [49]), P-protein (encoded by *GCV2* [50]), H-protein (encoded by *GCV3* [51]) and L-protein (encoded by *LPD1* [52]). Though the function of these proteins has not been confirmed experimentally, a similar function of the enzymes reported in *S. cerevisiae* indicates that these enzymes are highly conserved across fungal species. Thus, we analyzed the *GAT1* regulation of these genes by comparing transcriptional profiles of these genes in the two species using quantitative PCR (qPCR). In H99, expression of all four genes was significantly up-regulated when glycine was used as the sole nitrogen source compared to ammonium sulfate (Fig. 3A). In R265, however, such robust increases were not observed for the expressions of *GCV3* and *LPD1* (Fig. 3A). In H99, the addition of ammonium sulfate to the glycine media completely suppressed the expression of all 4 genes down to the levels observed with ammonium sulfate alone. However, in R265, the expression of *GCV1* and *GCV2* was only partially suppressed upon addition of ammonium sulfate (Fig. 3A). These results suggest that expression of two of the genes encoding glycine decarboxylase complex in R265 is not subject to the tight regulation by nitrogen catabolite repression as in H99.

We also examined the roles of *GAT1* in regulating the expression of glycine decarboxylase complex genes. Although expression of the four glycine decarboxylase genes in H99*gat1Δ* were induced when glycine was used as the sole nitrogen source, the induction levels of *GCV1, GCV2* and *GCV3* were not as high as in the wild type (*GCV1*: wt/*gat1Δ* = 1.91, p = 0.05; *GCV2*: wt/*gat1Δ* = 2.55, p = 0.01; *GCV3*: wt/*gat1Δ* = 1.8, p = 0.04, Fig. 3B). These data suggest that *GAT1* in H99 plays a role in derepressing the expression of these four genes and some factor(s) yet to be identified also regulates their induction when glycine is used as the sole nitrogen source. In R265*gat1Δ*, on the other hand, the expression levels of these four genes were close to wild type levels. These data suggest that the role of *GAT1* is less prominent in R265 than in H99 with respect to expressison of the four glycine decarboxylase genes when glycine is the sole nitrogen source.

### *GAT1* regulates virulence differently between *C. neoformans* and *C. gattii*

**Figure 4:**
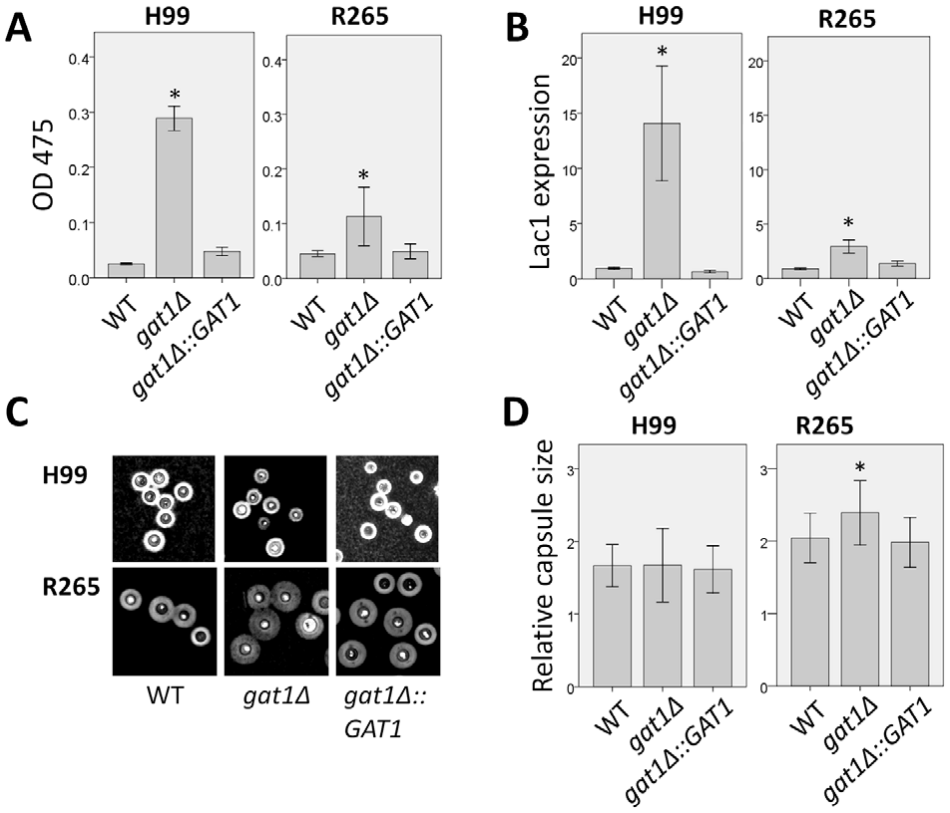
Disruption of *GAT1* enhanced melanin synthesis and *LAC1* expression in both species but capsule size increased only in R265. A) Cells were grown in melanin induction media using 10 mM proline as the nitrogen source. Melanin production was assayed by measuring the absorbance at 475 nm and B) *LAC1* expressions were quantified by real time PCR. C) Micrograph of cell grown in capsule induction media. D) The relative capsule size was measured in >100 cells for each strain grown in capsule induction media. Experiments were carried out in triplicates. * *p*≤0.05 by the student t-test. Error bars = 2 standard deviation.

**Figure 5:**
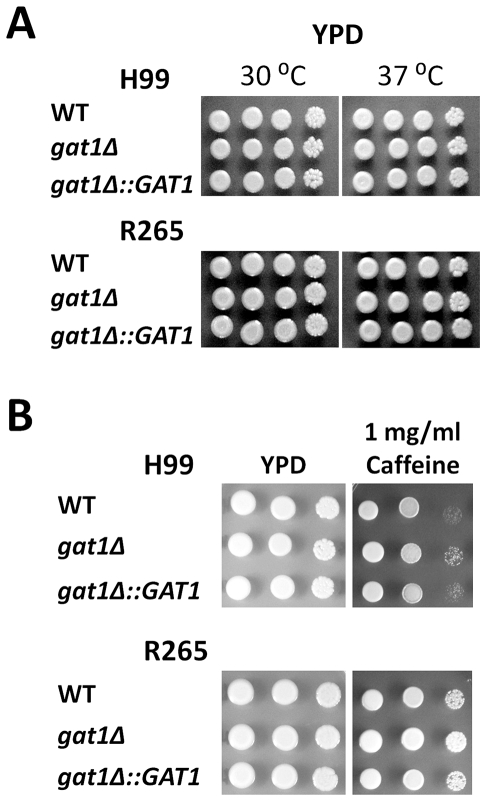
The role of *GAT1* in cell growth. A) *GAT1* is not associated with the growth rate of both species. Cell suspensions of 2 OD_600_ were spotted on YPD agar in three 10 fold dilutions and incubated for three days at the designated temperatures B) Similar spot assays were carried out on YPD with or without caffeine and plates were incubated at 30°C for 3 days. Experiments were carried out in duplicates.

**Figure 6:**
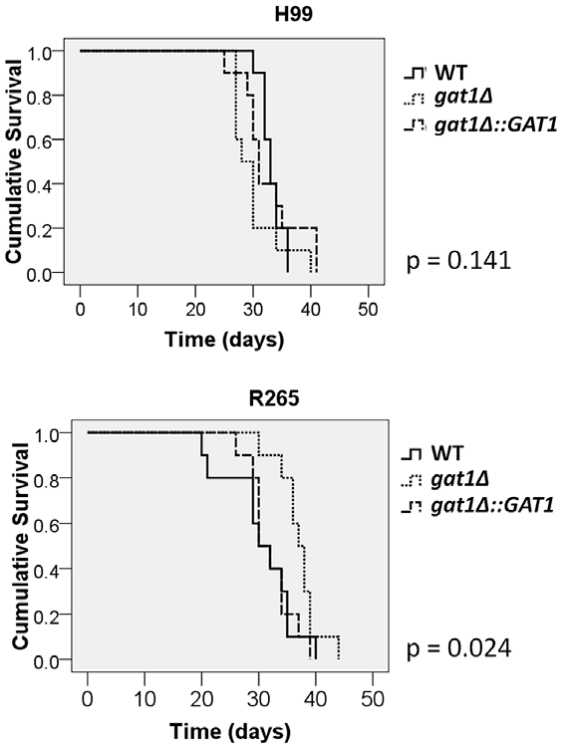
Virulence of *H99gat1Δ* was minimally enhanced while of *R265gat1Δ* was significantly reduced in murine inhalation model. 50,000 cells from each strain were inoculated intrapharyngeally to 10 Balb/c mice per group. Mice were fed with food and water ad libitum.

Two previous studies have shown that disruption of *GAT1* in H99 altered its virulence [35], [36]. We compared the impact of *GAT1* deletion in H99 and R265 on the expression of virulence factors in vitro including melanin and capsule production, ability to grow at 37°C and cell wall integrity in the presence of caffeine. Expression of the *LAC1* gene which encodes laccase responsible for melanin production [53] and the amount of melanin produced are important for the virulence in both species [43], [54]. The *gat1Δ* strains of both H99 and R265 exhibited an increase in melanin production (*gat1Δ*/wt = 10.26, *p* = 0.001 and 2.9, *p* = 0.023 for H99 and R265, respectively) and the expression of *LAC1* was found to be upregulated (*gat1Δ*/wt = 14.5, *p* = 0.001 and 3.2, *p* = 0.007 for H99 and R265, respectively) (Fig. 4A and 4B). When capsule production was compared in RPMI media with 5% CO_2_ at 37°C, only the *gat1Δ* strain of R265 but not of H99 produced a larger capsule than the wild type (*gat1Δ*/wt = 1.00, *p* = 0.915 and 1.17, *p* = 0.001 for H99 and R265, respectively) (Fig. 4C and 4D). However, neither R265*gat1Δ* nor H99*gat1Δ* showed any difference in growth rate at 37°C compared to wild type (Fig. 5A). However, a slight increase was observed in the tolerance to 1 mg/ml caffeine (Fig. 5B), a cell wall perturbing agent which has been used to test cell wall integrity of both species [43], [55]. Next, we compared the virulence between *gat1Δ* and wild type strains using a murine inhalation model. As reported previously, virulence of H99*gat1Δ* was slightly enhanced compared to H99 (*p* = 0.141, Fig. 6) [35], [36]. Surprisingly, however, virulence of R265*gat1Δ* was significantly more reduced than the wild type (*p* = 0.024, Fig. 6).

## Discussion

The ability to utilize specific nitrogen and carbon sources is among the standard methods used for the identification of yeast species [56], [57] as well as some filamentous fungi [58]. Differentiation of the two species in *Csc* also has been effectively carried out by both nitrogen and carbon utilization tests [24], [25], [26], [27], [47]. However, the efficacies of these biochemical tests were established long before molecular strain typing methods became available and the validity of these tests could have been compromised since the populations tested could contain strains of the same genotype. For example, previous studies have reported 100% [24] and 95% [25] specificity for D-proline utilization by *C. gattii* while we found a much lower specificity; only 80% of *C. gattii* strains yielded positive results in contrast to the 100% that were negative for *C. neoformans*. The CGB media, on the other hand, showed 100% specificity between the two species regardless of molecular type. Remarkably, the false negatives observed for D-proline utilization were found mainly among strains of the VGI and VGIII (5 negatives among 6 strains) molecular types. According to the phylogenetic analysis of global *Csc* strains based on multi gene sequence analyses, the VGI and VGIII molecular types formed a separate cluster with high bootstrap supports [6], [7]. Our results underscore the biological variation between different molecular types within *C. gattii*.

Upon screening for utilization of various amino acids as sole nitrogen sources, we discovered a new set of amino acids, phenylalanine, tryptophan and D-alanine, that could discriminate *C. neoformans* from *C. gattii* strains with reasonably high specificity. In fact, a previous study with much fewer numbers of strains also showed the discriminating power of phenylalanine and tryptophan utilization between the two species [35]. D-alanine has not been studied as the sole nitrogen source for differentiation of the two species. Our study showed that utilization of D-alanine has a higher discrimination power than that of D-proline, the second most commonly used diagnostic reagent next to CGB medium. Although rare, false negatives or questionable results on CGB media have been reported [25], [59], [60], [61] and these new sets of amino acid utilization panels can be applied to such rare strains. However, the strains with questionable CGB results might also yield ambiguous results with other nitrogen sources if the ambiguity is caused by a mutation in the genes associated with nitrogen metabolism. *C. gattii* strains that produce questionable CGB results can be tested for growth on tryptophan media in order to correct identify the questionable strains. Although molecular techniques enable confirmation of species identity, use of the one-step diagnostic media employing a species specific nitrogen source could be a cost effective option for developing countries with limited resources.

The nitrogen regulation by NCR in *C. neoformans* was studied based on the role of the GATA-type transcriptional activator Gat1 [35], [36]. As expected, Gat1 control of nitrogen regulation in the two species showed only minor differences. For example, Gat1 control of glycine utilizations in *C. gattii* was not as stringent as in *C. neoformans* while it was opposite in the utilization of aspartate. These results were supported by transcription analysis of the four genes encoding the glycine cleavage enzyme complex where the induction of these genes was positively controlled by Gat1 in *C. neoformans* but to a less extent in *C. gattii*. In addition, the NCR of glycine utilization in the presence of ammonium was clear-cut in *C. neoformans* but not in *C. gattii*. The difference in NCR of glycine utilization between the two species is reminiscent of creatinine metabolism where creatinine deiminase was inhibited by the accumulation of ammonium only in *C. neoformans* and not in *C. gattii*. As a result, growth of *C. neoformans* on creatinine media is considerably slower compared to *C. gattii* and creatinine bromthymol blue agar was the first one-step diagnostic media formulated to differentiate between the two species [47]. Moreover, it has been known that glycine metabolism is controlled by a complex process involving several genes [48], [62], [63]. For example, in *S. cerevisiae*, glycine can be synthesized from or converted to serine and threonine by serine hydroxymethyltransferase (Shm1 and Shm2) and threonine aldolase (Gly1). However, both species of *Csc* do not utilize threonine as effectively as other amino acids (Fig. 1) suggesting that the Gly1 role in glycine metabolism is minimal in *Csc* as has been reported in *Candida albicans* [64]. However, Shm1 may play a role in glycine metabolism in *Csc* since expression of *SHM1* was up-regulated 10 fold in both species when glycine was used as the nitrogen source instead of ammonium sulfate (data not shown). The nitrogen catabolic pathway, therefore, appears to be complex and further studies are needed to elucidate the pathways in *Csc*.

The ability to survive in a limited nutrient environment in the host is crucial for the growth of pathogenic organisms. For example, Gat1 influences virulence of several pathogenic fungi including, *Aspergillus fumigatus* and *C. albicans* [32], [33], [35], [36]. Our results on the virulence of H99*gat1Δ* is similar to the findings of Lee et al. [35] but different from that of Kmetzsch et al. [36]. Interestingly, we found that *gat1Δ* behaved differently between the two species in controlling the expression of some virulence factors *in vitro* and virulence in animals. It was surprising to observe that several virulence factors including capsule production, melanin synthesis and cell wall integrity, were negatively controlled by Gat1 in *C. gattii* while the virulence of R265*gat1Δ* was decreased in mice. In the host environment, the impact of impaired nitrogen utilization due to the lack of Gat1 function may be compensated by the increase in the virulence factors. The different impact of Gat1 in virulence between the two species might stem from the differences in their natural habitat and hence their pathobiology. It has been speculated that the virulence factors of the Cryptococcal species have evolved to combat their environmental predators such as soil amoeba and nematodes [65]. Pigeon droppings, the primary environmental niche of *C. neoformans*, is high in ammonium concentration and Gat1 may be constantly down-regulated while the primary natural habitat of *C. gattii* is detritus of trees which should be much lower in ammonium content than pigeon guano. This would have had a different impact on evolution of the role of Gat1 in regulation of virulence factors. However, whether the slight impact of Gat1 on the regulation of virulence factors observed in this study benefits survival of *Csc* in nature is unclear.

In conclusion, our study points out the different aspects of nitrogen metabolism between *C. neoformans* and *C. gattii*, two closely related etiologic agents of cryptococcosis. The range of nitrogen sources that can be used to differentiate between the two species has been expanded from previous studies to include a few more amino acids. The difference in nitrogen metabolism was confirmed by differences in nitrogen regulation by Gat1 and ammonium NCR which has a distinct effect on virulence between the two species. The difference in nitrogen utilization and its regulation by Gat1 shown in this study provides a compelling reason for characterization of the genes and metabolic pathways responsible for the differences. Global screening techniques namely transcriptome comparisons via cDNA microarrays and construction of *Agrobacterium tumefaciens* mediated insertional libraries [66] are currently being undertaken to elucidate the complex regulatory network of nitrogen metabolism in *Csc*.

## Materials and Methods

### Ethics Statement

The animal experiments were carried out with the approval and oversight of the Animal Care and Use Committee of the National Institute of Allergy and Infectious Diseases, United States National Institutes of Health, Bethesda MD.

### Strains and media

**Figure 7:**
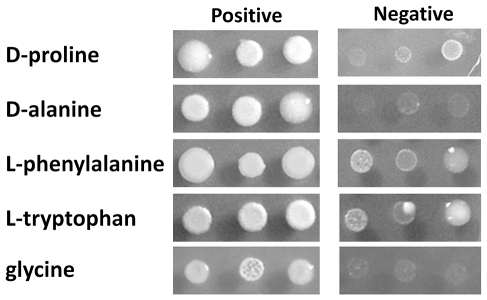
Examples of positive and negative growth on each nitrogen source. Cells were grown on 2% glucose YNB with 10 mM of each nitrogen source for 5–7 days at 30°C. 2 µl cell suspensions at an OD_600_nm of 0.02 were spotted on the media. Limitted growth on L-phenylalanine and L-tryptophan media was designated as negatives.

A global collection of 8 molecular types consisting of 31 *C. neoformans* and 36 *C. gattii* strains were revived from −80°C freezers of Molecular Microbiology Section, Laboratory of Clinical Infectious Diseases, NIAID, NIH, Bethesda, MD, USA and Molecular Mycology Research Laboratory, Westmead Hospital, Westmead, NSW, Australia. Molecular types of each strain were confirmed by *URA5*-RFLP as previously described (data not shown) [3] (Table 1). Cells were maintained on YPD agar (2% glucose, 1% yeast extract, 2% peptone and 2% Bacto agar) until use. For nitrogen utilization tests, cell suspensions were diluted to 0.02 OD600 nm for a single spot test or 10, 0.1 and 0.001 for serial dilution spot tests. The cell suspensions were spotted on 2% glucose YNB media (Becton, Dickinson and company, Sparks, MD, USA) with or without 10 mM of each indicated nitrogen source (Sigma-Aldrich, St. Louis, MO, USA) and incubated for 5–7 days at 30°C. For D-proline and D-alanine utilization tests, the results were recorded as negative when the growth was lower or equal to the growth on media without nitrogen source (Blank). For L-phenylalanine and L-tryptophan, results were recorded as negative when the strains grew poorly (see Fig. 7). CGB tests were carried out as described before and recorded as positive when the medium turns from yellow green to blue [27]. The nitrogen utilization tests were performed in duplicate or triplicate when differences were either evident or the experimental results were expected to fluctuate.

### Identification of gene orthologs

Protein sequences of each gene from *S. cerevisiae* genome project (http://www.yeastgenome.org/) were used to perform the BlastP in *C. neoformans* H99 genome project (http://www.broadinstitute.org/annotation/genome/cryptococcus_neoformans/MultiHome.html) and *C. gattii* R265 genome project (http://www.broadinstitute.org/annotation/genome/cryptococcus_neoformans_b/MultiHome.html). Genes were identified as follows (*C. neoformans*/*C. gattii*): *GAT1*– CNAG_00193/CNBG_0368, *GCV1* – CNAG_02818/CNBG_3472, *GCV2* – CNAG_01594/CNBG_3846, *GCV3* – CNAG_06316/CNBG_5135, *LPD1* – CNAG_02851/CNBG_5871.

### Gene disruptions and complementations

Disruption constructs of *GAT1* were created using overlapping PCR to link the 5′ and 3′ flanking region to nourseothricin antibiotic cassette [67]. The disruption constructs were transformed by biolistic transformation as described previously [68]. Transformants were selected on YPD containing nourseothricin. Homologous integrations were confirmed by PCR and southern hybridization. Disruptants were complemented by homologous integration of *GAT1* gene into *gat1Δ* using biolistic transformation. As none of *gat1Δ* was able to utilize L-serine as the sole nitrogen source, transformants grown on serine media (2% glucose YNB with 10 mM L-serine) were analyzed for the homologous complementations by PCR and confirmed by southern hybridization. (See table S1 for primers details)

### Quantitative real-time PCR

Strains were grown in YPD broth overnight to mid log phase. Cells were washed 3 times with 1× phosphate buffered saline (PBS: 137 mM NaCl, 2.7 mM KCl, 10 mM Na_2_HPO_4_, and 2 mM KH_2_PO_4_, pH 7.4) and re-suspended (1 OD600) in 2% glucose YNB supplemented with 10 mM of the specified nitrogen source. Cells were then grown at 30°C for 2 hrs and harvested for RNA extraction. Cell pellets were frozen and lyophilized. RNAs were extracted using RNA by Trizol® reagent (Invitrogen, Carlsbad, CA, USA) and cDNAs were generated by the Supercript III first-strand synthesis system (Invitrogen, Carlsbad, CA, USA) according to the manufacturer's protocol. Quantitative real-time PCR were performed using SYBR greener supermix (Invitrogen, Carlsbad, CA, USA) in Applied Biosystems 7500 Real-Time PCR System. Experiments were performed in triplicates using a relative standard curve method. (see Table S1 for primers details).

### Melanin production

Quantification of laccase activity for melanin synthesis was performed in triplicate according to the described method [69] with modification, using 0.1% glucose YNB without amino acid and ammonium chloride (Becton, Dickinson and Company, MD, USA) with 10 mM proline and 1 mM epinephrine (Sigma-Aldrich, MD, USA) at 30°C. L-proline was used as a nitrogen source due to its minimal growth reduction upon *GAT1* disruption [35]. Culture supernatant was obtained after 24 hours and assayed by measuring the absorbance at OD_475_nm using DU-64 spectophotometer (Beckman coulter, Fullerton, CA).

### Capsule formation

Amount of capsule produced was quantified by comparing the size ratio between cell with the capsules vs naked yeast (cell wall to cell wall diameter) as has been reported previously [43]. Briefly, each strain was grown overnight in YPD broth at 30°C. Yeast cells were harvested, washed and inoculated into capsule inducing RPMI with MOPS, HCO_3_, pH 7.3 and grown to a cell density of 10^6^–10^7^ cells/ml at 37°C with 5% CO_2_ for 72 hr [35], [70]. Capsule size of the cells stained with India ink was quantified by light microscopy.

### Ability to tolerate high temperature and cell wall perturbing agent

Strains were grown on YPD with or without 1 mg/ml caffeine, a known cell wall perturbing agent [43], [55] at 30°C or 37°C. Serial dilution spot test were carried out as described in the figure legends.

### Virulence in mice

BALB/c mice were inoculated with 50,000 yeast cells via intrapharyngeal inhalation as described [71], [72]. Briefly, cells were diluted in phosphate buffered saline (PBS) to 2.5×10^6^ cells/ml. Mice were anesthetized by isoflurane and partially hung by placing their incisors on a string while their lower back lie to a support patch. Tongues of the mice were gently held in full extension with padded forceps while a 20 µl suspension was pipetted onto the base of the tongue. Mice were allowed to breathe in the solution for 10 seconds. Tongues were then released and mice were placed in their cages to recover. Survival was monitored for 50 days.

### Statistical analysis

All statistical analyses specified were performed in SPSS 17.0 (IBM Inc., Armonk, New York).

## Supporting Information

Figure S1 **Examples of** ***GAT1/AREA*** **complementation which restored nitrogen utilization to the wild type level.** Wild type, *gat1Δ* and complemented strains were grown on 2% glucose YNB with 10 mM of each nitrogen source for 5–7 days at 30°C. . 5 µl of cells at OD_600_nm of 10, 0.1 and 0.001 were spotted on the media.
(TIF)

Table S1 **Primers details.**
(DOCX)
