# Supplementary figures and images for: Differences in Nitrogen Metabolism between Cryptococcus neoformans and C. gattii, the Two Etiologic Agents of Cryptococcosis
Source: PLoS One. 2012 Mar 27;7(3):e34258. doi: 10.1371/journal.pone.0034258 (PMC3313984; doi:10.1371/journal.pone.0034258)

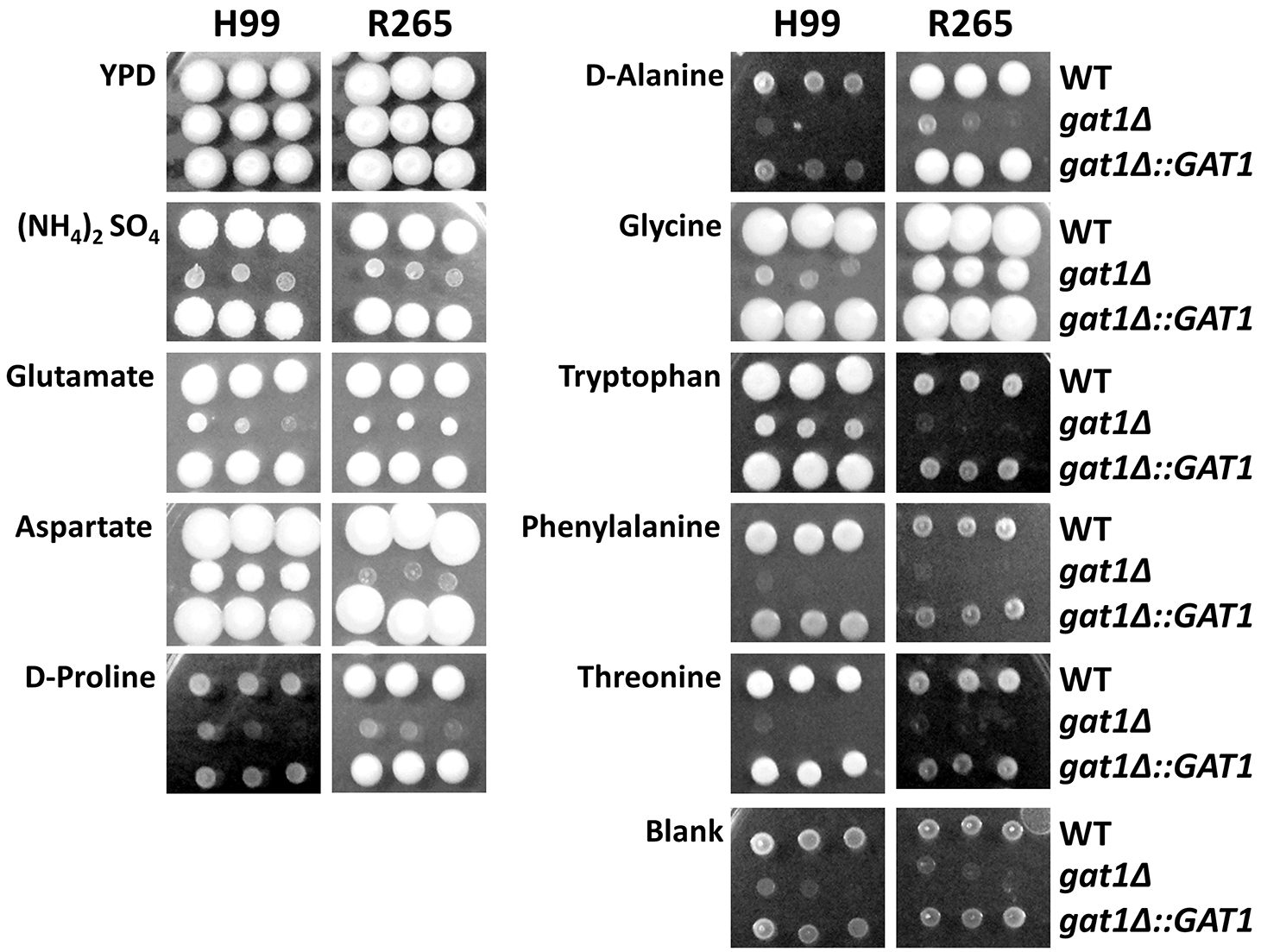

Supplement: Figure S1 — Examples of GAT1/AREA complementation which restored nitrogen utilization to the wild type level. Wild type, gat1Δ and complemented strains were grown on 2% glucose YNB with 10 mM of each nitrogen source for 5–7 days at 30°C. . 5 µl of cells at OD600nm of 10, 0.1 and 0.001 were spotted on the media. (TIF) [file pone.0034258.s001.tif]
