# Supplementary material for: Differences in Nitrogen Metabolism between Cryptococcus neoformans and C. gattii, the Two Etiologic Agents of Cryptococcosis
Source: PLoS One. 2012 Mar 27;7(3):e34258. doi: 10.1371/journal.pone.0034258 (PMC3313984; doi:10.1371/journal.pone.0034258)
Supplement: Table S1 — Primers details. (DOCX) [file pone.0034258.s002.docx]

**Table S1.** Primers details

| **Name** | **5' - 3' sequences** | **Description** |
| --- | --- | --- |
| PAI3F | ATAAGCATGCAGGATTCGAGTG | Amplification of *NAT* resistance cassette |
| PAI3R | TCGTGGTTTCAGAGACAAGGAG | Amplification of *NAT* resistance cassette |
| HGATF1 | CCTAACCAATCCCGCAATTGTA | Deletion of H99 *GAT1* |
| HGATF2 | CTCCTTGTCTCTGAAACCACGATGAATGAGCGAATGAATGGA | Deletion of H99 *GAT1* |
| HGATR1 | ATGGGTATCGAGCATACGAGGA | Deletion of H99 *GAT1* |
| HGATR2 | CACTCGAATCCTGCATGCTTATCATGGAAGCTTGTCCATCCT | Deletion of H99 *GAT1* |
| HGATEF | CACACACTTCTTCCCCATCCAT | Deletion of H99 *GAT1* |
| RGATF1 | GTTGGCGGCTATGTATGAGGTC | Deletion of R265 *GAT1* |
| RGATF2 | CTCCTTGTCTCTGAAACCACGAGAGGGGAGATTCGGAGAGAG | Deletion of R265 *GAT1* |
| RGATR1 | TATTTACGCCGTACCCCAACTG | Deletion of R265 *GAT1* |
| RGATR2 | CACTCGAATCCTGCATGCTTATCATGGAAGCTTGTCCATCCT | Deletion of R265 *GAT1* |
| RGATEF | AGGGATAATAAGGGCCGACAGA | Deletion of R265 *GAT1* |
| HACT1RTF2 | AGTGTCTGGATCGGTGGTTC | Quantitative PCR for H99 *ACT1* |
| HACT1RTR2 | ACTTTCGGTGGACGATTGAG | Quantitative PCR for H99 *ACT1* |
| HGCV1RTF | GTCTCGAAGCCGGTATGTGT | Quantitative PCR for H99 *GCV1* |
| HGCV1RTR | CGCGCCTATAATCTCAAAGC | Quantitative PCR for H99 *GCV1* |
| HGCV2RTF | ACCCGACTTTTCCAAGAGGT | Quantitative PCR for H99 *GCV2* |
| HGCV2RTR | CAATCCTGGAACAGGGAAGA | Quantitative PCR for H99 *GCV2* |
| HGCV3RTF | TGTCGTCTTTGTCGAGTTGC | Quantitative PCR for H99 and R265 *GCV3* |
| HGCV3RTR | AGAGCTTGACCTTGCAGAGC | Quantitative PCR for H99 and R265 *GCV3* |
| HLPD1RTF | GTCTCGAAGCCGGTATGTGT | Quantitative PCR for H99 *LPD1* |
| HLPD1RTR | CGCGCCTATAATCTCAAAGC | Quantitative PCR for H99 *LPD1* |
| HGAT1RTF | GTTCGCCAACGAGTGAAAAT | Quantitative PCR for H99 *GAT1* |
| HGAT1RTR | GGGAAGGGTTGCTTTCTTTC | Quantitative PCR for H99 *GAT1* |
| HLAC1RTF | GGAAAGTTGGCTGTGGTTGT | Quantitative PCR for H99 *LAC1* |
| HLAC1RTR | CAAATGCATTGGGATCAGTG | Quantitative PCR for H99 *LAC1* |
| RACT1RTF2 | CACCATTGGTAACGAACGA | Quantitative PCR for R265 *ACT1* |
| RACT1RTR2 | AGAACCACCGATCCAGACAC | Quantitative PCR for R265 *ACT1* |
| RGCV1RTF | CAGACTGTCGCTGTTTCCAA | Quantitative PCR for R265 *GCV1* |
| RGCV1RTR | ATTTTCCAGGGAAAGCAGGT | Quantitative PCR for R265 *GCV1* |
| RGCV2RTF2 | TGCACCATGAAGCTCAACTC | Quantitative PCR for R265 *GCV2* |
| RGCV2RTR2 | GGTGACAAGGGACAAGTCGT | Quantitative PCR for R265 *GCV2* |
| RLPD1RTF3 | CGTACGACGTGGTCATCATC | Quantitative PCR for R265 *LPD1* |
| RLPD1RTR3 | GCATTGCTTTGGAAGGGATA | Quantitative PCR for R265 *LPD1* |
| RGAT1RTF | GACCAGGTACACCGACGAGT | Quantitative PCR for R265 *GAT1* |
| RGAT1RTR | CTGCTCGGTTCCTTTTCTTG | Quantitative PCR for R265 *GAT1* |
| RLAC1RTF | ACCTTCATGGCAACGAGTTC | Quantitative PCR for R265 *LAC1* |
| RLAC1RTR | ACAACCACAGCCAACTTTCC | Quantitative PCR for R265 *LAC1* |
